# Opening of Astrocytic Mitochondrial ATP-Sensitive Potassium Channels Upregulates Electrical Coupling between Hippocampal Astrocytes in Rat Brain Slices

**DOI:** 10.1371/journal.pone.0056605

**Published:** 2013-02-13

**Authors:** Jiangping Wang, Zhongxia Li, Mei Feng, Keming Ren, Guoxia Shen, Congying Zhao, Xiaoming Jin, Kewen Jiang

**Affiliations:** 1 Department of Neurology, The Children’s Hospital Zhejiang University School of Medicine, Hangzhou, Zhejiang, China; 2 Department of Rehabilitation, The Children’s Hospital Zhejiang University School of Medicine, Hangzhou, Zhejiang, China; 3 Stark Neurosciences Research Institute Indiana University School of Medicine, Indianapolis, Indiana, United States of America; 4 Department of Laboratory, The Children’s Hospital Zhejiang University School of Medicine, Hangzhou, Zhejiang, China; University of Texas Medical Branch, United States of America

## Abstract

Astrocytes form extensive intercellular networks through gap junctions to support both biochemical and electrical coupling between adjacent cells. ATP-sensitive K^+^ (K_ATP_) channels couple cell metabolic state to membrane excitability and are enriched in glial cells. Activation of astrocytic mitochondrial K_ATP_ (mitoK_ATP_) channel regulates certain astrocytic functions. However, less is known about its impact on electrical coupling between directly coupled astrocytes *ex vivo*. By using dual patch clamp recording, we found that activation of mitoK_ATP_ channel increased the electrical coupling ratio in brain slices. The electrical coupling ratio started to increase 3 min after exposure to Diazoxide, a mitoK_ATP_ channel activator, peaked at 5 min, and maintained its level with little adaptation until the end of the 10-min treatment. Blocking the mitoK_ATP_ channel with 5-hydroxydecanoate, inhibited electrical coupling immediately, and by 10-min, the ratio dropped by 71% of the initial level. Activation of mitoK_ATP_ channel also decreased the latency time of the transjunctional currents by 50%. The increase in the coupling ratio resulting from the activation of the mitoK_ATP_ channel in a single astrocyte was further potentiated by the concurrent inhibiting of the channel on the recipient astrocyte. Furthermore, Meclofenamic acid, a gap-junction inhibitor which completely blocked the tracer coupling, hardly reversed the impact of mitoK_ATP_ channel's activation on electrical coupling (by 7%). The level of mitochondrial Connexin43, a gap junctional subunit, significantly increased by 70% in astrocytes after 10-min Diazoxide treatment. Phospho-ERK signals were detected in Connexin43 immunoprecipitates in the Diazoxide-treated astrocytes, but not untreated control samples. Finally, inhibiting ERK could attenuate the effects of Diazoxide on electrical coupling by 61%. These findings demonstrate that activation of astrocytic mitoK_ATP_ channel upregulates electrical coupling between hippocampal astrocytes *ex vivo*. In addition, this effect is mainly via up-regulation of the Connexin43-constituted gap junction coupling by an ERK-dependent mechanism in the mitochondria.

## Introduction

Astrocytes in the mammalian central nervous system form extensive intercellular networks through gap junctions [1]–[3]. These gap junctions allow direct cytoplasmic continuity [4]–[7], and they are postulated to support biochemical and electrical coupling between adjacent cells.

The spatial organization of astrocytes in monolayer culture is simple whereas their three-dimensional network *ex vivo* renders a more natural state of neurobiological function and complexity for research [8]–[10]. Recent studies have begun to address the *in situ* intercellular communication of astrocytes in the nucleus accumbens [11] and the hippocampus [12], [13]. Data from these studies have shed light on the properties of astrocytic electrical coupling *ex vivo* under physiological and pathological conditions. Despite the critical role of electrical coupling in this network, the regulatory mechanisms behind this gap junction-mediated or -supported electrophysiological condition remain largely unknown.

ATP-sensitive potassium (K_ATP_) channels are heteromultimer complexes of subunits from members of the inwardly rectifying K^+^ channels and the ATP-binding cassette protein superfamilies. K_ATP_ channels couple metabolic state to membrane excitability, and thus they participate in a variety of physiological functions [14], [15]. Moreover, in the nervous system, K_ATP_ channel activation is involved in the control of neuronal excitability [16]–[18] and seizure propagation [14], [19]–[24]. Given the importance of astrocytes on brain function [25] and the enrichment of K_ATP_ channel in glial cells, K_ATP_ channels might be responsible for some critical activities of astrocytes or at least play a role in them [26]–[28]. More recently, it has been revealed that activation of astrocytic K_ATP_ channels, particularly the mitochondrial K_ATP_ (mitoK_ATP_) channels, affects glutamate uptake and astrocytic activation [29]–[32]. However, whether mitoK_ATP_ channel possesses a regulatory effect on electrical coupling between directly coupled astrocytes in brain slices has not been investigated yet.

We and other groups have demonstrated previously that activation of astrocytic mitoK_ATP_ channels enhances gap junctional coupling and reverses neurotoxin-induced dysfunction of astrocytic coupling both in astrocytic cultures and brain tissues [33], [34]. However, blocking gap junction with meclofenamic acid (MFA) do not inhibit the electrical coupling between directly coupled astrocytes in hippocampal slices [13]. Given the causal link between astrocytic mitoK_ATP_ channel activity and gap junction function, and the conflicting data on gap junction's role in electrical coupling led us to investigate the effects of astrocytic mitoK_ATP_ channels on this gap junction-mediated/supported electrical coupling.

In this study, we addressed the following issues: 1) whether mitoK_ATP_ channels directly regulate the electrical coupling between directly coupled astrocytes, 2) whether blocking of gap junctions affects mitoK_ATP_ channel's regulation of astrocytic electrical coupling, and 3) the possible mechanisms underlying this astrocytic mitoK_ATP_ channel-induced electrical coupling. We found that activation of astrocytic mitoK_ATP_ channel increased the electrical coupling ratio in rat brain slices while blockage of the channel immediately induced an inhibition of the electrical coupling. Accordingly, the latency time of transjunctional currents was shortened by 50% following channel activation. When activation of mitoK_ATP_ channels in one astrocyte was combined with inhibition of that in its recipient pair cell, the electrical coupling ratio was further elevated significantly. Meanwhile, MFA, the gap junction inhibitor which completely blocked the tracer coupling, failed to impair the electrical coupling and counteract the effect of activated mitoK_ATP_ channel on it. When the mitoK_ATP_ channel was activated in astrocytes, phospho-ERK was detected in gap junctional subunit immunoprecipitates. Finally, inhibiting ERK could attenuate the effects of activation of mitoK_ATP_ channels on electrical coupling. Our findings suggest that astrocytic mitoK_ATP_ channel regulates on gap junctional coupling through multiple mechanisms including direct electrical coupling via gap junctions, ion buffering, and metabolic machinery.

## Materials and Methods

### Ethics Statement

All animal procedures were complied with the guidelines of the Animal Advisory Committee at Zhejiang University.

### Hippocampal slice preparation

Hippocampal slices were prepared from male Sprague-Dawley rats aged 21 to 25 days (referred to as P21), as previously described [13]. Briefly, rats were deeply anesthetized with diethyl ether in a chamber before decapitation, and their brains were removed from the skulls and placed in an ice-cold, oxygenated (5% CO_2_/95% O_2_) slice preparation solution containing (in mM): 26 NaHCO_3_, 1.25 NaH_2_PO_4_, 2.5 KCl, 10 MgCl_2_, 10 glucose, 0.5 CaCl_2_, 240 sucrose. Coronal slices of 300 µm thickness were obtained using a Vibroslicer (Leica VT 1000) and sections containing the hippocampus were selected, as described previously (Zhou et al., 2006, 2009). Slices were transferred to a nylon holder basket (AutoMate Scientific) immersed in artificial cerebral spinal fluid (aCSF) containing (in mM): 125 NaCl, 25 NaHCO_3_, 10 glucose, 3.5 KCl, 1.25 NaH_2_PO_4_, 2.0 CaCl_2_, and 1 MgCl_2_ at room temperature (20–22 °C). For the recovery period, slices were incubated in aCSF at 33 °C for∼ 60 min. This was followed by incubation at 22 °C for ∼60 min, and then the slices were transferred to the recording chamber and superfused (3 ml min^−1^) with aCSF at room temperature. All solutions were saturated with 95% O_2_/5% CO_2_.

### Electrophysiology

Astrocytes located in the hippocampal CA1 region were visualized with an infrared-sensitive CCD camera with a ×63 water-immersion lens (ZEISS, Examiner A1) and recorded using whole-cell techniques (MultiClamp 700B Amplifier, Digidata 1440A analog-to-digital converter) and pClamp 10.2 software (Axon Instruments/Molecular Devices). Based on the cellular morphology under IR-DIC and the electrophysiological characteristics, we can differentiate astrocytes from neurons or NG2 glia (Table S1). To avoid any cell-to-cell variation in coupling strength due to location shift, the astrocytes studied were 50–70 µm beneath the slice surface. Single or dual patch whole-cell currents were sampled at 10–20 kHz, filtered at 1–2 kHz. The recording pipettes were fabricated from borosilicate capillaries (OD: 1.5 mm; Warner Instrument, Hamden, CT) using a Flaming/Brown Micropipette Puller (Model P-97; Sutter Instrument, Novato, CA). The pipettes had a resistance of 3.5–5.5 MΩ when filled with a KCl-based pipette solution containing (in mM): 140 KCl, 0.5 Ca_2_Cl, 1.0 MgCl_2_, 5 EGTA, 10 HEPES, 3 Mg-ATP, and 0.3 Na-GTP (pH = 7.3, 290 ± 5 mOsm). We only performed whole-cell recordings if the initial seal resistance was or above 2 GΩ. The membrane potential (*V_M_*) was read in the ‘‘*I* = 0’’ mode. The membrane capacitance (*C_M_*), membrane resistance (*R_M_*), and access resistances (*R_a_*) were measured using the ‘‘Membrane test’’ program. Test data was excluded if the depolarized resting membrane potential exceeded −75 mV or the access resistance (Ra) exceeded 15 MΩ or varied for more than ± 5 MΩ during recording.

Mature hippocampal astrocytes characteristically showed an exceedingly low membrane resistance of ∼2 MΩ, which caused a large voltage error of ∼80% in the whole-cell voltage clamp recording. Because the voltage error cannot be effectively improved by *R_a_* compensation [35], we did not compensate for the *R_a_* in this study and applied command voltage (*V_c_*) in all the *I*–*V* plots instead.

To measure the electrical coupling between directly coupled astrocytes, dual patch recording was performed on two candidate astrocytes spaced 20∼40 µm apart and located on the same focal plane of the slice. A series of 50 ms voltage pulses ranging from −240 mV to +80 mV in increments of 20-mV was applied to the stimulated cell (S_cell_) while the corresponding cell (recipient cell, R_cell_) was constantly voltage-clamped at −80 mV, a voltage close to the average resting membrane potential of astrocytes. Usually, bidirectional transjunctional currents were recorded for both astrocytes. The latency time of the transjunctional currents were determined by measuring the time lag between each maximum rise slope's appearance of S_cell_ and R_cell_.

To address the effects of astrocytic mitoK_ATP_ channel on the electrical coupling between directly coupled astrocytes, we used two agents, Diazoxide (DIZ) and 5-hydroxydecanoate (5-HD) at a working concentration of 100 µM and 300 µM respectively, or both in aCSF and/or internal solution. ASCF containing meclofenamic acid (MFA), an inhibitor of gap junction, was used to examine the effect of gap junction blockage on electrical coupling and mitoK_ATP_ channel's regulation on this functional blockage. Preliminary work has shown a time-dependent run-down of electrical coupling in recordings utilizing normal aCSF solution [13], which is most likely caused by frequent stimulation during the coupling ratio measurement. Therefore, less frequent measurements (on 1-min interval) and short-term (10-min) treatments were used in this study, and each treatment was followed by a 10-min recovery in normal aCSF solution.

To control the exact duration of the direct drug application for an individual astrocyte during dual patch clamp, we first formed a GΩ seal for each paired cell. We let the seal stabilize for 1–2 min, and then ruptured the two cells consecutively within 15 s.

### Differential interference contrast image capture and high-speed two-photon measurement of interastrocytic distance

DIC images of astrocytes in slices were acquired during electrophysiological recording using an IR1000 camera (DAGE-MTI). The interastrocytic distance was defined as the distance between the two soma centers of the selected astrocytes and was measured using the scale bar on the video monitor.

Intraastrocytic loading of Lucifer yellow (LY, 0.1%) or Alexa Fluor® 594 (60 µM) in brain slices was done as previously described [36]. The loading time was 10 min for both dyes. In some dual patch experiments, electrode solutions containing only LY or Alexa Fluor® 594 were used to determine the cross diffusion of LY and Alexa Fluor® 594 within recorded pairs. High-speed two-photon imaging was performed by using a custom-built two-photon microscope based on Ti:Sapphire pulsing laser (model: Chameleon, repetition rate: 80 MHz, pulse width: 140 fs; Coherent, USA) and a resonant galvo-mirror (8 kHz; GSI) system. The scanner was mounted on an upright microscope (BX51WI, Olympus, Tokyo, Japan) equipped with a water-immersion objective lens (60x/0.8 Olympus, Japan). Emitted photons were detected by two detection channels equipped with photomultiplier tubes (H7422-40; Hamamatsu), a “green” channel for LY-generated fluorescence (480–560 nm) and a “red” channel for Alexa-594-generated fluorescence (580–680 nm). Full-frame images at 514×514 pixel resolution were acquired at 30 Hz by custom-programmed software based on LabVIEW (National Instruments, USA). At the end of each experiment, a Z-stack of the fluorescently labeled astrocyte was acquired (0.8 µm step size). For each slice, 100 - 150 z-plane images were typically acquired to include the entire LY-stained syncytia spanning a thickness of 100−120 µm in the slice. Three-dimensional projection was generated by ImageJ software. To determine the distance between neighbor astrocytes, the 3D territories of surrounding astrocytes loaded with LY were determined according to the staining. Rotating the 3D images of these LY-loaded astrocytes allowed visual identification of the nearest neighbor astrocytes that were directly coupled to the recorded cell based on the criterion that no additional cell body and/or astrocytic processes could be seen between them and the soma of the recorded cell. The number of directly coupled astrocytes and the distance from them to the recorded cell were obtained following image reconstruction by ImageJ software. During two-photon imaging of the cross diffusion within cell pairs, no bleed-through of emission signals between LY and Alexa FLuor® 594 channels was detected.

### Immunocytochemistry and confocal image acquisition

For immunofluorescent staining, the slices were first fixed immediately (4% Paraformaldehyde) after removal from recording chamber at 4 °C for 24 h. Fixed sections were treated with 3% (vol/vol) normal donkey serum in PBS containing 0.5% Triton X-100 for 1 h and then incubated with rabbit anti-glial fibrillary acidic protein (GFAP) antibody (1∶500, Abcam) and mouse monoclonal anti-NG2 (1∶500, CHEMICON International) at 4 °C for 36–48 h. Immunoreactivity was visualized using FITC488, Cy3 or Cy5-conjugated donkey anti-rabbit or mouse IgG (1∶1000, VECTOR Laboratories) based on the dye introduced into the pipette solution during the recording test. After immunofluorescence examination, slices were transferred onto a glass-bottom chamber containing PBS for confocal image acquisition using a Carl Zeiss LSM510 confocal microscope. The intensity of LY or Alexa Fluor® 594 signals and the location of marks made by recording electrodes were used to distinguish the recorded cells from the other coupled cells. Co-labeling of LY or Alexa Fluor® 594 with GFAP or NG2 was determined by the LSM Image Browser.

### Immunoblotting for ERK, JNK and Connexin (Cx) 43

Astrocytes and fractions of membrane and mitochondria were prepared as described previously [33], [37] (see detail in Text S1).

Samples were electrophoresed on a 12.5% polyacrylamide gel and then blotted onto a polyvinylidene difluoride (PVDF) membrane (Millipore). Blots were incubated with a blocking solution containing Tris buffer with 5% nonfat dry milk and 0.1% Tween 20, and then with antibodies against ERK1/2 (1∶1000), phospho-ERK1/2 (1∶500), JNK (1∶1000), phospho-JNK (1∶600) and Cx43 (1∶800) (Cell Signaling Technologies). Secondary antibodies used were peroxidase-linked anti-rabbit IgG F(ab’)2 fragments (1∶2000) (Amersham Biosciences). Proteins in the blots were visualized using an ECL Western blotting detection kit (Amersham Biosciences), and band intensity was quantified by ImageJ. Data were converted to relative optical density as percentages with glyceraldehyde-3-phosphate dehydrogenase (GAPDH) (1∶5000, Sigma) designated as 100%.

### Immunoprecipitation (IP) of Cx43

To investigate whether Cx43 physically interacts with ERK, protein fractions from DIZ-treated astrocytes and from untreated control astrocytes were immunoprecipitated using an anti-Cx43 antibody (BD Transduction Laboratories). In brief, 1000 µg protein was solubilized by 500 µl of IP buffer (20 mM Tris–HCl (pH 7.4), 1 mM EGTA, 5 mM NaN_3_, 50 mM NaCl, 1 mM PMSF, 50 mM Na_3_VO_4_, 1% Triton X-100, 0.5% NP-40 and a protease inhibitor cocktail) and preincubated with 50 µl protein G magnetic beads (New England BioLabs) for 1 h to remove proteins that can bind non-specifically to the beads. The supernatant was incubated with 5 µg of anti-Cx43 antibodies (1∶800) for 1 h, and this mixture was incubated with 50 µl of fresh beads for 1 h. A magnetic field was applied to this IP mixture, and the supernatant was removed. The beads were washed 2 times using 500 µl of IP buffer, re-suspended in 30 µl of SDS sample loading buffer [125 mM Tris-HCl (pH 6.8), 4.3% SDS, 30% glycerol, 10% h-mercaptoethanol, 0.01% bromophenol blue], and incubated at 70 °C for 5 min. Finally, 20 µl of the supernatant was used for immunoblotting by anti-phospho-ERK1/2 antibody (1∶500) (Cell Signaling Technologies). PVDF membranes were then stripped using a Re-Blot Western Recycling Kit (CHEMICON International) and used for re-blotting with anti-ERK1/2 antibody (1∶1000) (Cell Signaling Technologies).

### Data analysis

The electrical coupling ratio was calculated using this equation: Coupling ratio  =  (*I*_Rcell_ / *I*_Scell_)×100%, in which the *I*_Scell_ is the whole-cell current measured from the S_cell_, and *I*_Rcell_ represents the transjunctional current measured from the R_cell_. Data were presented as mean ± SEM. Statistical significance for pre- and post-treatment difference in each experimental group was determined by Student's two-tailed t-test. Results from different experimental groups were analyzed by one-way ANOVA test. Multiple comparisons were made with Tukey's Honestly Significant Differences test. Experimental values were considered significant if *P*<0.05.

## Results

### Distances between directly coupled astrocytes in hippocampal CA1 region

Astrocytes in a single syncytium can be coupled directly (nearest neighbors) or indirectly via intermediate astrocyte(s). We conducted a morphometric analysis to determine how the intensity of cell coupling varied with intercellular distance among directly coupled astrocytes. Astrocytic syncytia in the CA1 stratum radiatum region were loaded with LY through whole-cell recording electrodes, and 10 min after intracellular loading, the syncytia was visualized by two-photon 3D imaging. The number of LY loaded cells in 12 syncytia varied from 42 to 96 with an average of 79 ± 15 cells (mean ± standard deviation). In each slice, the recorded astrocyte was used as the reference cell to measure the interastrocytic distances of other astrocytes coupled directly to it (Fig. S1A). The results showed that an average of 12±4 astrocytes was directly coupled to a single astrocyte at an average interastrocytic distance of 43.5±12.4 µm (135 measurements from 12 syncytia). The protoplasmic astrocytes were almost spherical in shape and had an average diameter of 55.6 µm. The majority (92%) of directly coupled cells (the nearest neighbors) was located within an interastrocytic range of 30–60 µm (Fig. S1B).

Furthermore, we found that the electrical coupling ratio progressively declined with increasing interastrocytic distance in P21 rats, but remained relatively stable when the interastrocytic distance was 20–40 µm (Fig. S2). We also noticed a less variable *R_m_* (1.1 to 3.8 MΩ) among astrocytes in P21 rats that are within the same interastrocytic distance range (Fig. S3) [13]. Because a higher coupling ratio was always detected in astrocytes with a higher *R_m_* as the S_cell_ (n = 3, data not shown), the paired astrocytes with a variant *R_m_* (> 1 MΩ) were excluded from our studies.

### Activation of mitoK_ATP_ channels increased the electrical coupling of directly coupled astrocytes in rat hippocampal slices

**Figure 1:**
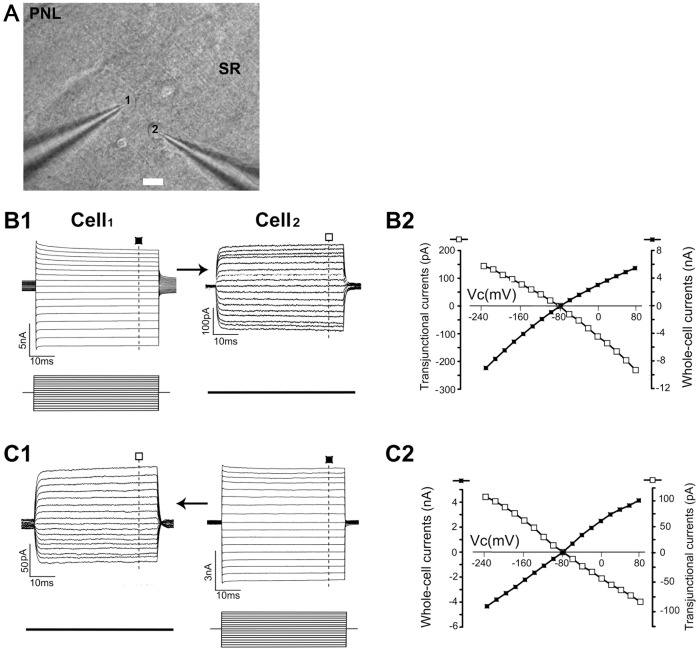
Electrical coupling between directly coupled astrocytes. (**A**) DIC image of CA1 stratum radiatum region, showing the placement of dual electrodes for two astrocytes spaced 35 µm apart (marked as ‘‘1’’ and ‘‘2’’) (bar  = 10 µm). SR, stratum radiatum; PNL, pyramidal neuron layer. In (**B1,2**) and (**C1,2**), whole-cell voltage steps (from -240 to 80 mV in 20-mV increments for 50 ms) were sequentially delivered to the stimulated cell (S_cell_) Cell1 (**B1**) and Cell2 (**C1**), respectively. The recipient cell (R_cell_) was constantly held at −80 mV. The measured transjunctional currents from each voltage step was subtracted from the amount of basal currents for construction of the *I-V* plots presented in **B2** and **C2**. The graph of transjunctional currents (□) shows a linear *I-V* relationship that is of opposite polarity and proportional to the delivered voltage steps (▪).

For dual patch recording, two candidate astrocytes spaced 20–40 µm apart were chosen for electrical coupling measurements (Figs. 1A, S2, S3). The bidirectional transjunctional currents were recorded (Fig. 1B, C were obtained from the recording pair of astrocytes shown in Fig. 1A). The polarity of the transjunctional currents in the R_cell_ was opposite to that of voltages delivered to the S_cell_ (*I*–*V* plots shown in Fig. 1B2, C2). To exclude the possibility that the currents recorded in the R_cell_ are currents leaking out of the S_cell_ and spreading through the extracellular space to the recipient astrocyte, the electrode from the S_cell_ soma was withdrawn after dual patch recording and then moved back to its original position without forming a gigaohm seal. Using this procedure the command voltages in the S_cell_ did not induce any transjunctional currents in the R_cell_ (n = 5). Furthermore, we tested the whole-cell voltage pulses at △ ± 160 mV for 10 s and found no evidence of any voltage- and time-dependent inactivation component of transjunctional currents (n = 10; data not shown), indicating a series of voltage pulses applied in our experiments is sensitive to detect the electrical coupling between astrocytes. Finally, we did not detect any electrical coupling between paired astrocyte-neuron (Fig. S4A1; n = 10), astrocyte-NG2 glia (Fig. S4A2, B; n = 20), and astrocyte-interneuron (n = 5; data not shown).

We first examined the time course of the effect of astrocytic miyoK_ATP_ channel on electrical coupling in the presence of (1) DIZ, a mito-K_ATP_ channel opener alone, (2) 5-HD, a mito-K_ATP_ channel inhibitor alone, or (3) DIZ + 5-HD. We measured electrical coupling at a 1-min interval immediately after slices were exposed to the treatment. Each treatment lasted for 10 min and was followed by a 10-min recovery through perfusion of normal aCSF solution.

**Figure 2:**
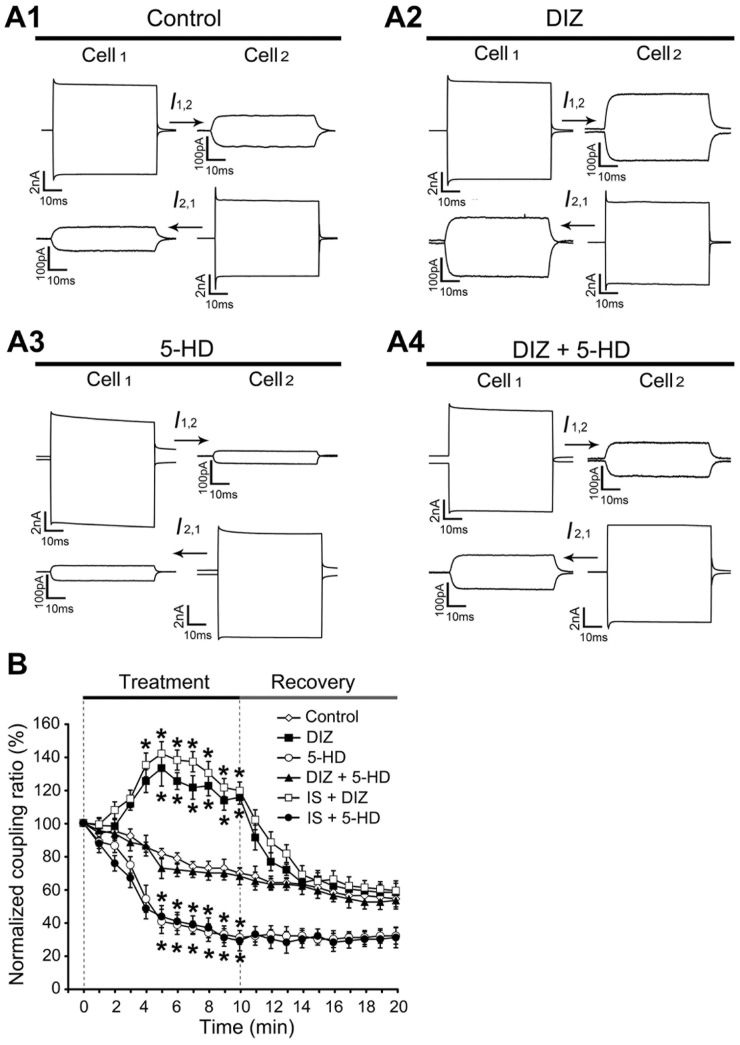
Activation of mitoK_ATP_ channels increased the electrical coupling of directly coupled astrocytes in slice. (**A**) Representative recordings of a recorded astrocyte pair after addition of DIZ, 5-HD, or both for 5 min. The coupling ratio was increased with DIZ but decreased with 5-HD. (**B**) Time course of changes in the electrical coupling ratio under the following conditions: control (n = 16), DIZ (n = 13), 5-HD (n = 8), DIZ+5-HD (n = 9), internal solution containing DIZ (IS + DIZ, n = 10), and internal solution containing 5-HD (IS + 5-HD, n = 8). The duration for each treatment was 10 min, followed by a 10-min recovery in control aCSF solution. The increased electrical coupling ratios, already apparent 3 min after exposure to DIZ, reached peak level at 5 min, and then lasted with little adaptation till the end of treatment. Blocking the astrocytic mito-K_ATP_ channel with 5-HD immediately inhibited electrical coupling, and after 10 min, the coupling was inhibited to 71% of the initial control level. The coupling ratio with DIZ/5-HD treatment was the same as that with control in the 10-min duration. Also, we did not find any difference in the coupling ratios between slices treated with an internal solution containing astrocytic mitoKATP channel modulator (DIZ or 5-HD) and the DIZ or 5-HD treated slices respectively, during the recording period (0 to 10 min). All the paired astrocyte recordings were obtained in P21–25 rats and from the CA1 stratum radiatum region. Mean ± SEM; * *P*<0.05 compared to the control. DIZ, Diazoxide; 5-HD, 5-hydroxydecanoate.

We found that activation of astrocytic mito-K_ATP_ channel increased the electrical coupling ratios 3 min after exposure to DIZ. This impact reached the peak level by 5 min and then lasted with little adaptation till the end of treatment (Fig. 2A, B). Blocking the channel with 5-HD induced an immediate inhibition of electrical coupling, resulting in a decrease of 71% of the initial control level by the end of the 10-min treatment. Changes in inhibition were not detected in the group receiving DIZ/5-HD. Even though we modified the protocol of stimulation to include less frequent measurements (on 1-min interval) and short-term (10-min) treatments, there was still a run-down trend of the control, which was most likely caused by frequent stimulation during the coupling ratio measurement.

When coupling ratios among the four groups at each time point were compared using multiple comparison tests, no significant difference was detected between the control and DIZ/5-HD group at any time point during or after treatment. In addition, the differences in the coupling ratios among DIZ, control, and 5-HD groups became significant after 5 min of treatment (indicated by * in Fig. 2B), but this trend disappeared right after the recovery procedure started. While the coupling ratio of the 5-HD group remained at a low level throughout the 10-min recovery, it took almost 3 min for the DIZ group to have its ratio return to base level during washing. However, no statistical difference was observed among the groups from the very beginning of recovery.

### Activation of mitoK_ATP_ channels increased the speed of transjunctional current in slices

**Figure 3:**
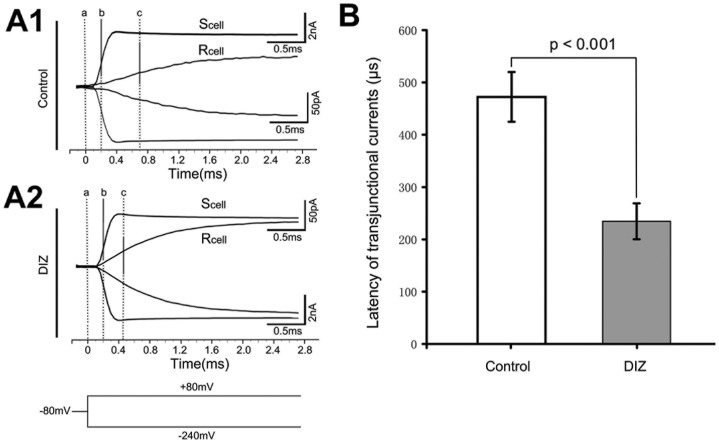
Activation of mitoK_ATP_ channels decreased the latency time of the transjunctional currents in slice. (**A**) Currents in S_cell_ induced by a pair of ±160 mV voltage steps and the transjunctional currents received by the R_cell_. The holding potential for both cells was -80 mV. ‘‘a’’ indicates the onset time point of the voltage steps. The time of the maximal current rise slope from both the S_cell_ (b - a) and R_cell_ (c - a) were calculated using the Clampfit 10.2 software. The latency of the transjunctional currents is defined as [(c - a) - (b - a)]. Representative recording trace of a recorded astrocyte pair after incubation with DIZ for 6 min (**A2**). The control experiment is shown in (**A1**). (**B**) Bar graph shows that at 6 min after exposure to DIZ, the latency time was decreased by 50% with an average of 234.54±34.43 µs (n = 13). These results were determined from paired astrocyte recordings with interastrocytic distances of 20 – 40 µm. Mean ± SEM; DIZ, Diazoxide; R_cell_, recipient cell; S_cell_, stimulated cell.

The latency time of the transjunctional currents was determined by measuring the interval between the maximum rise slope appearances between the S_cell_ and R_cell_, which is an indicator of the speed of the transjunctional current from S_cell_ to R_cell_. This measurement was obtained from paired astrocytes at an interastrocytic distance of 20 - 40 µm. At 6 min after exposure to DIZ, activation of astrocytic mitoK_ATP_ channel decreased the latency time by 50% of the control at an average of 234.54 ± 34.43 µs (Fig. 3, n = 13).

### Activation of mitoK_ATP_ channels in a single astrocyte increased the electrical coupling of directly coupled astrocytes in slices

Previous reports have demonstrated that gap junctional coupling was neuronal activity-dependent [38]. Thus, treating the slice with DIZ would also open the neuronal K_ATP_ channels, and this would induce hyperpolarization of the neurons, consequently influencing the gap-junctional coupling. In order to eliminate this effect on activated K_ATP_ channels by neurons, we used an internal solution containing the channel modulators. We inserted pipettes into target cells in slices under a low positive pressure to prevent leakage of the modulators, and increased the perfusion volume of aCSF in the recording chamber to immediately remove any modulators that leaked.

Compared with what we observed in DIZ or 5-HD treated slices, we did not detect any differences in the coupling ratios when we used an internal solution containing the same astrocytic mitoK_ATP_ channel modulator (DIZ or 5-HD) during the recording period respectively (0 to 10 min, Fig. 2B).

**Figure 4:**
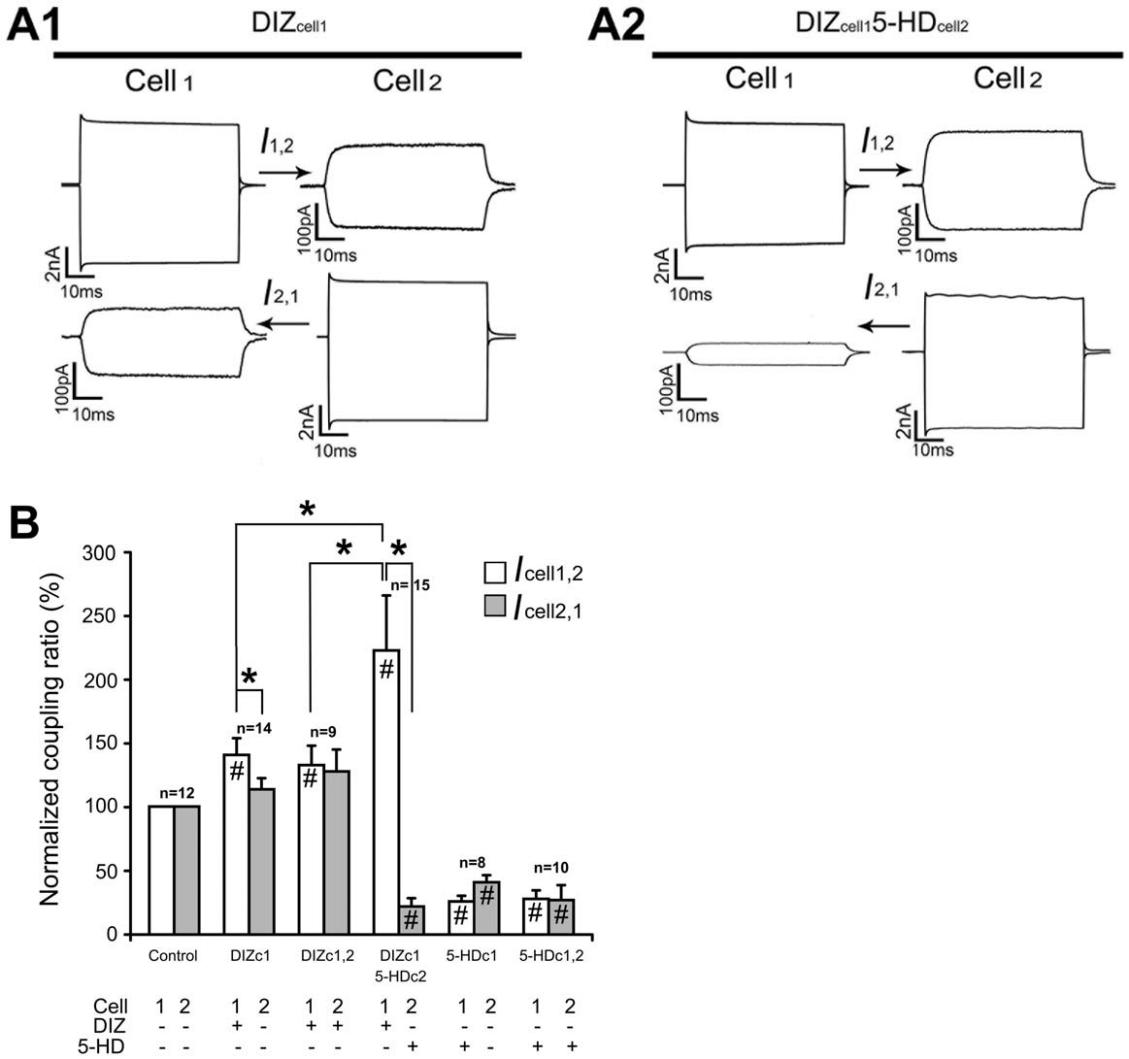
Activation of mitoK_ATP_ channels in single astrocyte increased the electrical coupling of directly coupled astrocytes in slices. Internal solution containing test drugs was used to diminish the pharmacological effects induced by opening/blocking neuronal K_ATP_ channels in slice. (A) Representative electrical coupling trace of a recorded astrocyte pair after addition of DIZ in cell1 (A1), and after addition of DIZ in cell1 and 5-HD in cell2 for 5 min (A2). (B) Bar graph shows that compared to that observed in DIZ-treated slices, we did not find any differences in coupling ratios with the internal solution containing DIZ at 5 min. Interestingly, the level of coupling ratios from the S_cell_ with DIZ-contained internal solution (S_cell-DIZ_) to the R_cell_ with normal internal solution (R_cell-_) was higher than that from the S_cell_ with normal internal solution (S_cell-_) to the R_cell_ with DIZ-containing internal solution (R_cell-DIZ_). Moreover, the level of coupling ratios from the S_cell-_ to the R_cell_ with 5-HD-containing internal solution (R_cell-5-HD_) was higher than that from the S_cell_ with 5-HD-containing internal solution (S_cell_-_5-HD_) to the R_cell-_, even though the differences in the ratios were not significant. When S_cell-DIZ_ was combined with R_cell-5-HD_ in our experiments, this elevation in coupling ratios became significant, as the level increased by 58% and 68% than that from S_cell-DIZ_ to R_cell-_ and S_cell-DIZ_ to R_cell-DIZ_ respectively. These results were determined from paired astrocyte recordings at interastrocytic distances of 20 – 40 µm. Mean ± SEM; * *P*<0.05. ^#^ *P*<0.05 compared to the control. DIZ, Diazoxide; 5-HD, 5-hydroxydecanoate; R_cell_, recipient cell; S_cell_, stimulated cell.

Interestingly, we observed that the coupling ratio from the S_cell_ with DIZ-containing internal solution (S_cell-DIZ_) to the R_cell_ with normal internal solution (R_cell-_) was higher than that from the S_cell_ with normal internal solution (S_cell-_) to the R_cell_ with DIZ-containing internal solution (R_cell-DIZ_) (Fig. 4A1 and B). On the other hand, the coupling ratio from the S_cell-_ to the R_cell_ with 5-HD-containing internal solution (R_cell-5-HD_) was higher than that from the S_cell_ with 5-HD-containing internal solution (S_cell_-_5-HD_) to the R_cell-_, but the difference was not significant. When S_cell-DIZ_ was combined with R_cell-5-HD_, this change in coupling ratio became significant (Fig. 4A) as the level was higher than that from S_cell-DIZ_ to R_cell-_ and S_cell-DIZ_ to R_cell-DIZ_ by 58% and 68% respectively (Fig. 4B).

### The gap junction inhibitor MFA was unable to completely block the mitoK_ATP_ channels’ effects on electrical coupling of directly coupled astrocytes

**Figure 5:**
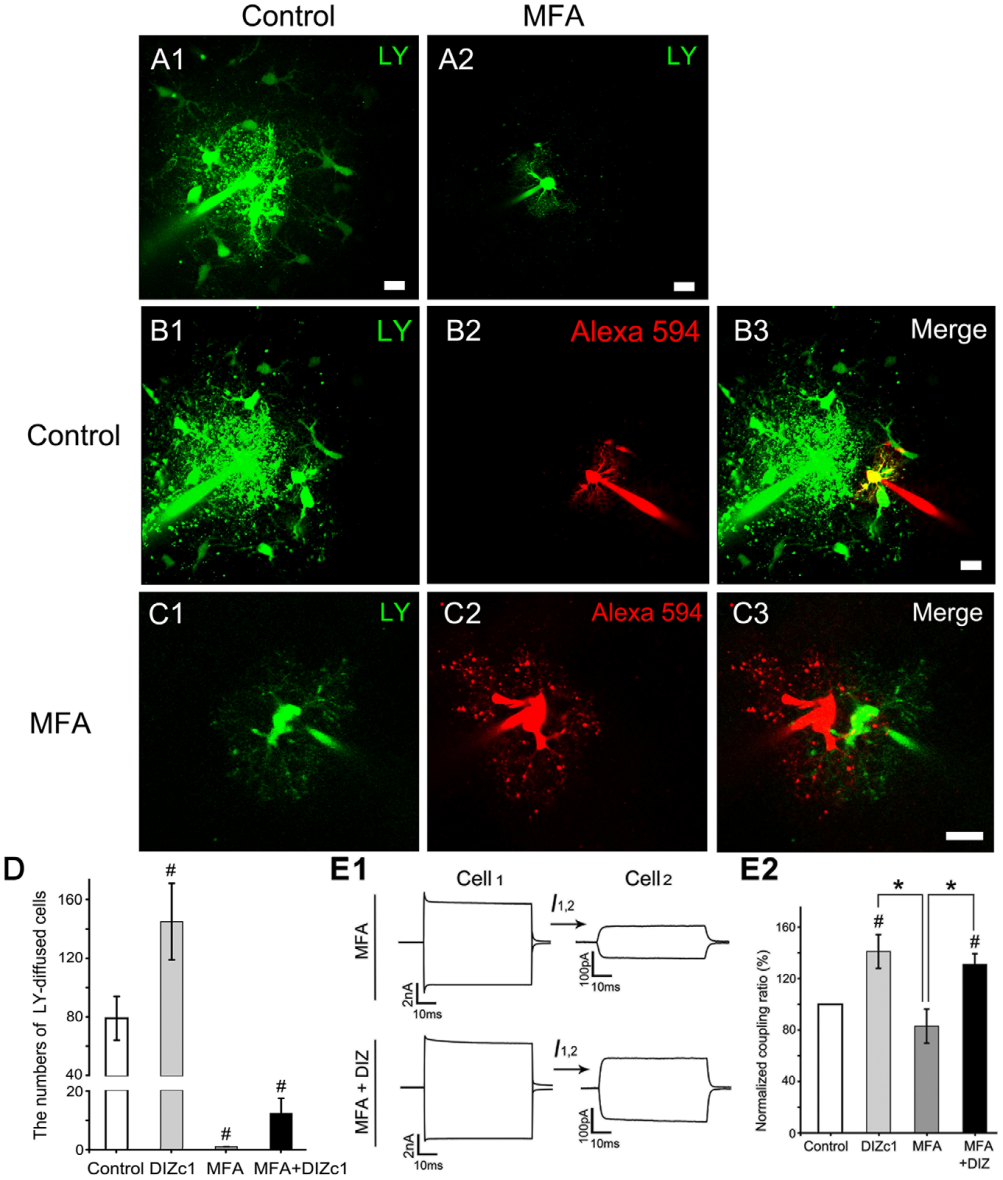
MFA, a gap junction inhibitor, blocked the tracer coupling but not electrical coupling and was unable to block the mitoK_ATP_ channels’ effects on electrical coupling of directly coupled astrocytes. The Loading of LY into cells for 10 min resulted in transcellular diffusion (A1, bar  = 10 µm) but not when cells were pre-treated with MFA (A2, bar  = 20 µm). Furthermore, the separate loading of LY (green, C1) and Alexa Fluor® 594 (red, C2) into cells via dual patch recording electrodes for 10 min also did not lead to transcellular diffusion of the two tracers upon pre-treatment of the slices with MFA 1 h prior to recording. However, when the slices were not pre-treated with MFA, we observed transcellular diffusion of LY (green, B1) but not Alexa Fluor® 594 (red, B2) (bar  = 10 µm). (D) Bar graph shows that DIZ significantly increased the transcellular diffusion of LY (DIZc1, n = 9), MFA inhibited the transcellular diffusion of LY (n = 7), and DIZ increased the transcellular diffusion of LY even after pre-treatment of the slices with MFA 1 h prior to recording (n = 10). (E1) Representative electrical coupling trace of recorded astrocyte pairs after incubation with DIZ for 5 min under conditions with or without MFA pre-treatment. (E2) Bar graph shows that MFA inhibited the coupling ratio by 17% (n = 8), while at 5 min after loading the cell with DIZ, the MFA inhibited the coupling ratio only by 7% (n = 12). These results were determined from paired recordings from astrocytes at interastrocytic distances of 20 – 40 µm. Mean ± SEM; * *P*<0.05. ^#^ *P*<0.05 compared to the control. MFA, Meclofenamic acid; DIZ, Diazoxide; 5-HD, 5-hydroxydecanoate.

We pre-treated the slice with 100 µM MFA, a gap junction inhibitor, for 1 h before recording, and found that MFA inhibited the coupling ratio by 17% (n = 8) (Fig. 5E2). However, this difference was statistically insignificant which was consistent with the results from previous reports [13]. We also tested two other gap junction inhibitors (carbenoxolone and octanol) and obtained similar results (data not shown). However, MFA did completely inhibit LY and Alexa Fluor® 594 transfer between astrocytes in hippocampal slices [36]. As shown in Fig. 5A1, loading of LY into the cells for 10 min resulted in transcellular diffusion. This was not observed when cells were pre-treated with MFA (Fig. 5A2). Furthermore, transcellular diffusion of two tracers (LY (green) and Alexa Fluor® 594 (red)) loaded via recording electrode for 10 min was blocked when the slices were pre-treated with MFA for 1 h [Fig. 5C (MFA) vs. Fig. 5B (control)]. We also measured transcellular diffusion of LY, and found that DIZ significantly increased the diffusion [29], [33]. Blockage of gap junctions with MFA completely inhibited this transcellular diffusion. Importantly, when the slices were treated with MFA for 1 h, DIZ enhanced the LY diffusion, although not to a significant degree (Fig. 5D).

We then used the electrode internal solution containing DIZ to test whether the gap junction inhibitor MFA can block the mitoK_ATP_ channels’ effects on electrical coupling. At 5 min after loading the astrocyte with DIZ, MFA did not eliminate DIZ induced-elevation on the coupling ratio (Fig. 5E). The application of MFA inhibited the effects of mitoK_ATP_ channel activation on electrical coupling by 7% (n = 12, Fig. 5E), considerably less than the coupling ratio of the DIZc1 group (also shown in Fig. 4B).

### DIZ may regulate astrocytic electrical coupling via up-regulation of the mitochondrial Cx43-constituted gap junction coupling by an ERK-dependent mechanism

**Figure 6:**
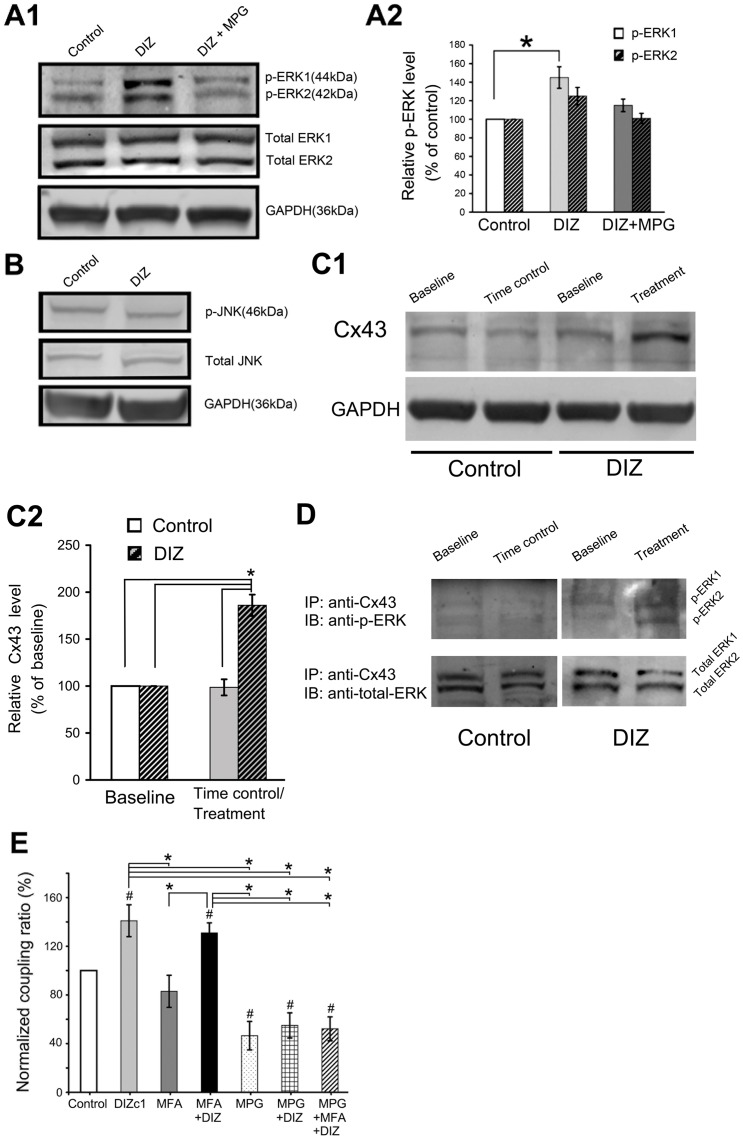
DIZ may regulate astrocytic electrical coupling via up-regulation of Cx43 constituted gap junction coupling through an ERK-dependent mechanism. (**A1**) Representative immunoblots of lysates from untreated control, DIZ-treated, and DIZ + MPG-treated astrocytes. (**A2**) The level of ERK phosphorylation (p-ERK1) was significantly increased by DIZ, though for the level of JNK was unchanged (**B**). The increase in the level of phosphorylated ERK after treatment with DIZ was suppressed by treatment with MPG. n = 4. (**C1**) Representative immunoblots of CX43 in untreated control and in DIZ-treated astrocytes. (**C2**) A summary of densitometry data normalized by baseline level in each group. DIZ significantly increased the level of Cx43. n = 4. *p<0.05. (**D**) Immunoblotting for phospho-ERK in Cx43-immunoprecipitated samples. Phospho-ERK was detected in Cx43-immunoprecipitates in samples treated with DIZ. The results from three experiments were the same. DIZ = 100 µM diazoxide, p-ERK  =  phosphorylated ERK, p-JNK  =  phosphorylated JNK, MPG  =  1 mM N-2-mercaptopropionyl glycine, Cx43  =  Connexin 43, IP  =  immunoprecipitate, IB  =  Immunoblotting. (**E**) MPG, a synthetic aminothiol antioxidant that could suppress ERK1/2 phosphorylation, attenuated the effects of DIZ on electrical coupling. We pre-treated the slice with MPG for 1 h before recording, and found that MPG inhibited the coupling ratio by 54% (n = 6) and diminished the DIZ-nduced coupling ratio by 61% (n = 8). When combining inhibition of ERK1/2 with blockage of gap junction, the DIZ-induced electrical coupling was diminished by 63% (n = 7). Mean ± SEM; * *P*<0.05. ^#^ *P*<0.05 compared to the control. DIZ, Diazoxide; GAPDH, glyceraldehyde-3-phosphate dehydrogenase; MFA, Meclofenamic acid; MPG  =  1 mM N-2-mercaptopropionyl glycine.

Because 100 µM-DIZ is a selective mitoK_ATP_ channel opener, its activation of astrocytic mitoK_ATP_ channel increased the electrical coupling ratio, and gap junction inhibitors barely inhibited the DIZ-induced effects on electrical couplings, we studied the possible mechanisms underlying this astrocytic mitoK_ATP_ channel-induced electrical coupling by immunoblotting and IP based on the available literature. Treatment with 100 µM DIZ increased the levels of phosphorylated ERK1 and ERK2 by 1.5 and 1.3-fold, respectively (Fig. 6A), but did not significantly change the level of phospho-JNK in the mitochondria (Fig. 6B). The Levels of total ERK1/2 and JNK did not significantly change after DIZ treatment (data not shown, Fig. 6AB). DIZ-induced ERK1/2 phosphorylation was suppressed by N-2-mercaptopropionyl glycine (MPG), a synthetic aminothiol antioxidant (Fig. 6A). The levels of total ERK1, ERK2, phospho-JNK and total JNK did not change significantly after DIZ treatment with or without MPG (data not shown). Treatment with MPG alone did not modify phospho-ERK1/2 level (data not shown).

The level of mitochondrial Cx43 in astrocytes significantly increased by 70% after 10-min DIZ treatment (Fig. 6C1 and C2). As shown in Fig. 6D, ERK was coimmunoprecipitated with Cx43, but no phospho-ERK was detected in untreated control samples. However, in the DIZ-treated astrocytes, phospho-ERK was detected in Cx43 immunoprecipitates. On the other hand, the level of total ERK coimmunoprecipitated with Cx43 was similar between DIZ-treated and -untreated samples.

The levels of phosphor-ERK1/2, total ERK1/2, phospho-JNK and total JNK in the membrane fractions did not change significantly following DIZ treatment with or without MPG (data not shown). Furthermore, the level of Cx43 did not change following 10-min DIZ treatment, and no phospho-ERK signal was detected in the Cx43 immunoprecipitates following DIZ-treatment (data not shown).

Finally, we test whether inhibiting ERK1/2 would attenuate the effects of DIZ on electrical coupling. We pre-treated the slice with MPG, a synthetic aminothiol antioxidant to suppress ERK1/2 phosphorylation for 1 h before recording, and found that MPG inhibited the coupling ratio by 54% (n = 6) (Fig. 6E). Further more, MPG indeed inhibited the DIZ-induced coupling ratio by 61% (n = 8) (Fig. 6E). When combining inhibition of ERK1/2 with blockage of gap junction, the DIZ-induced electrical coupling was diminished by 63% (n = 7, Fig. 6E). It seems that there is no cumulative effect by this combination.

## Discussion

### What is the contribution of the plasmalemmal membrane K_ATP_ (sK_ATP_) channel in the mitoK_ATP_ channel regulated-electrical coupling of directly coupled astrocytes?

K_ATP_ channels are located in various parts of the cell including the surface of the plasmalemmal membrane (sK_ATP_) [39]–[42]. Manipulation of the K_ATP_ channel status in various cellular types and at various intracellular locations has revealed the various functions of K_ATP_ channel [39], [43]–[46]. We have demonstrated that blocking of the sK_ATP_ channels could increase the gap junctional coupling. We also found that blocking the sK_ATP_ channels increased the electrical coupling, but only accounted for 6 percent (using tolbutamide, a sK_ATP_ channel blocker) of the enhancement induced by total blockage of membrane K^+^ channels (tolbutamide plus barium, Ba^2+^, which was referred to as an inhibitor of most membrane K^+^ channels; data not shown). In the current study, we found that activation of mitoK_ATP_ channels increased the electrical coupling ratios. We postulate that mitoK_ATP_ channel regulators mimic the role of ATP on the sK_ATP_ channels, which suggests that intracellular energy status of the astrocytes might regulate intercellular communication through gap junctions. Activation of mitoK_ATP_ channels could sustain its own metabolic machinery [47] and the subsequent increase in ATP would inhibit the sK_ATP_ channels, which would result in an increase in gap junctional permeability [48]. However, if inhibition of mitoK_ATP_ leads to the compromised energy reserve of the astrocytes [47], the sK_ATP_ channels would be activated, resulting in a decrease in gap junctional permeability and vice versa. However, sK_ATP_ channels might be only responsible for a small part of the induced elevation of electrical coupling attributable to mitoK_ATP_ channel activation because the coupling ratio when mitoK_ATP_ channels were activated was 2.5 folds higher than when sK_ATP_ channels were inhibited. Furthermore, the increase in the speed of transjunctional current observed upon activation of mitoK_ATP_ channels in slices, suggests that elevation in electrical coupling occurs through other mechanisms. In addition, we have evidence showing that the resting membrane current in recorded astrocytes is not changed after addition of 100 µM DIZ (Fig. S5), and our IP results demonstrate that DIZ acts directly through mitochondrial Cx43, but not membrane Cx43. Consequently, DIZ, a selective mitoK_ATP_ channel opener, would not affect the resting membrane currents in astrocytes.

### Electrical coupling of directly coupled astrocytes is distinct from gap junctional tracer-coupling

An increase in the electrical coupling by Ba^2+^ was demonstrated in slices [13] which indicates that inhibition of membrane K^+^ channels might allow for leakage of injected currents out of the cell before the currents reached the gap junctions. Gap junction-mediated coupling might only be responsible for a small portion of the electrical coupling ratios, as we found that blocking the gap junctions completely inhibited the tracer-coupling but not the electrical coupling which was only reduced by 17%. Another possible explanation is that tracer-coupling might not be a precise coupling indicator for the gap junctional coupling. However, the fact that complete tracer uncoupling of hippocampal astrocytes was shown in mice lacking both Cx30 and Cx43 do not support this hypothesis [49]. On average, at least for the CA1 stratum radiatum of P21 Sprague–Dawley rats, each astrocyte couples to 12 nearest neighbors and shows a trend of increasing coupling percentages at larger interastrocytic distances. For example, the measured ratios of 0.29, 0.65, 1.59, 2.45, 3.17 and 3.85 pairs corresponded to interastrocytic distances of 0–10.0, 10.1–20.0, 20.1–30.0, 30.1–40.0, 40.1–50.0, and 50.1–60.0 µm, respectively. Using the exponential equation shown in Fig. S2, we calculated a total accumulative coupling ratio in the range of 19–26.4% at the gap junctional interface between the recorded astrocyte and 12 directly coupled astrocytes (half interastrocytic distance), 5.7–10.7% at the distance between adjacent soma centers of 12 directly coupled cells (full interastrocytic distance), and 0.8–2.2% at the periphery of the tier one astrocytes (one and one-half interastrocytic distance). These calculations indicate that the command voltages will essentially disappear beyond the first shell of astrocytes around the recorded astrocyte. However, both distances and efficiencies of the tracer-coupling were far more than of electrical coupling (Fig. 5A1 and S4).

### Relationship between astrocytic mitoK_ATP_ channels and gap junctions

Astrocytic mitoK_ATP_ channels and gap junctions were functionally coupled together. We and other groups have demonstrated that activation of astrocytic mitoK_ATP_ channels in astrocytes enhanced both the gap junctional coupling and protein expression level of Cxs. Furthermore, activation of astrocytic mitoK_ATP_ channels reversed the neurotoxin-induced impairment of astrocytic coupling and down-regulation of Cx expression, both in astrocytic cultures and in brain tissues [33], [34]. In the current study, we showed that activation of mitoK_ATP_ channels enhanced the electrical coupling; in contrast, blocking mitoK_ATP_ channels substantially inhibited electrical coupling in slices. Even though gap junction-mediated coupling might be responsible for only a part of the electrical coupling ratios as discussed above, a study that examined K_ATP_ channel function on islets and beta-cells in transgenic mice found that a small percentage of K_ATP_ channel activity in the beta-cells is sufficient to maintain strong glucose-dependent functions, but that inhibition of gap junctions causes a complete loss of K_ATP_ channel activity that controls membrane potential [50]. This supports our conclusion that K_ATP_ channels and gap junctions might influence each other synergistically.

### Does astrocytic K_ATP_ channels regulate the electrical coupling of directly coupled astrocytes for spatial buffering of K^+^ *in vivo*?

The role of astrocytes in homeostatic functions is well established. One of the key mechanisms proposed underlying the regulation of K^+^ levels by astrocytes is the spatial redistribution of K^+^ termed spatial buffering [4], [51]. Disruption in astrocytic gap junction-regulated K^+^ buffering is involved in many pathologic conditions [52]–[54]. Interestingly, a large capacity for K^+^ clearance is conserved in mice with coupling-deficient astrocytes, indicating that gap junction-dependent processes only partially account for K^+^ buffering [53]. Astrocytic gap junction-regulated K^+^ buffering is also believed to remove K^+^ by spatial buffering, possibly through inwardly rectifying potassium channels that enable uptake and redistribution of extracellular K^+^ [55]. In the current study, we showed in slices that activation of mitoK_ATP_ channels enhanced the electrical coupling and gap junctional tracer coupling. Although only 1.6% of the locally elevated K^+^, assumed to occur at the astrocytic soma, can travel across gap junctions in each astrocyte pair, the fact that each astrocyte couples to 12 another astrocytes in CA1 striatum radiatum indicates a collective dispersion of 20.7–24.2% of K^+^ to the nearest neighbor astrocytes [13]. Therefore, regulation of the electrical coupling of directly coupled astrocytes by astrocytic K_ATP_ channels might partially be for spatial buffering of K^+^ *in vivo*. Further study using double-barreled K^+^-selective/reference microelectrodes combined with pharmacological experiments in slices are needed to clarify this question [56].

### Proposed model of astrocytic mitoK_ATP_ channel-induced electrical coupling

**Figure 7:**
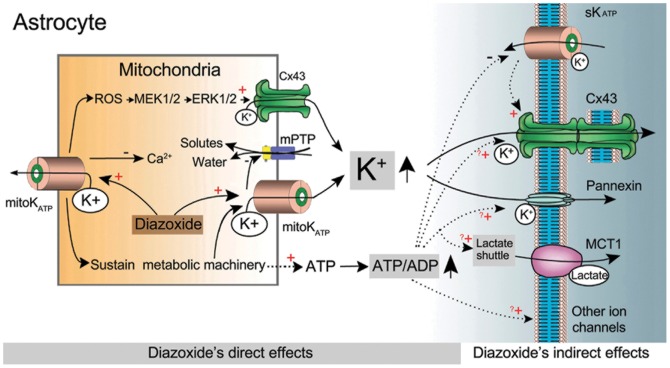
Proposed model of astrocytic mitoK_ATP_ channel-induced electrical coupling. Schematic drawing depicting a potential mechanism for astrocytic mitoK_ATP_ channel-induced electrical coupling based on the present study and available literature. Opening of astrocytic mitoK_ATP_ channels by DIZ activates ERK by a ROS-dependent mechanism, and increases direct interactions between mitochondrial Cx43 and ERK, which results in an increased permeability of mitochondrial gap junctions for K^+^. Opening of mitoKATP channels also allows the mitochondrial K^+^ influx into the soma. Both may increase the K^+^ buffering via sK_ATP_ channels and the membrane gap junction coupling. Activation of astrocytic mitoK_ATP_ channels sustains metabolic machinery and increases the ATP/ADP ratios, which inhibits the inward K^+^ currents of sK_ATP_ channels. In addition, the elevated levels of ATP, proposed to be able to promote astrocytic multiple functions including gap junction coupling, lactate transportation and ion transferring, may facilitate the electrical coupling. Abbreviations: Cx43, Connexin 43; MCT1, monocarboxylase transporter 1; mitoK_ATP_, mitochondrial ATP-sensitive potassium channel; mPTP, mitochondrial permeability transition pore; ROS, reactive oxygen species; sK_ATP_, membrane ATP-sensitive potassium channel.

To explain the current findings, we propose a model for the mechanism underlying astrocytic mitoK_ATP_ channel-induced electrical coupling (Figure 7). Importantly, we showed that the DIZ-induced electrical coupling could be diminished by 7% by blocking gap junctions (Fig. 5E), indicating that membrane gap junctional coupling plays a partial role in electrical coupling between two directly coupled astrocytes. Because 100 µM-DIZ is a selective mitoK_ATP_ channel opener [57], and its activation of astrocytic mitoK_ATP_ channel could up-regulate the Cx43-constituted gap junction coupling in the mitochondria by an ERK-dependent mechanism (Fig. 6), this DIZ-induced electrical coupling may not directly be mediated by membrane gap junctions. Blocking gap junction with MFA resulted in complete tracer uncoupling but did not significantly affect electrical coupling (Fig. 5). Other mechanisms may be responsible for electrical coupling by 83%. This hypothesis can be further tested using hippocampal astrocytes in mice lacking both Cx30 and Cx43 [49]. In addition, as discussed above, results from both theoretical and experimental studies suggest that regulation by astrocytic K_ATP_ channels of electrical coupling of directly coupled astrocytes might be for spatial buffering of K^+^ *in vivo*, and therefore, even small changes in the concentration of the underlying substrates (K^+^) may change the electrical coupling. Obviously, regulation of the astrocytic mitoK_ATP_ channels will change the concentration of the cytoplastic K^+^, and we propose that this change will regulate the electrical coupling. Indeed, we found that activation of astrocytic mitoK_ATP_ channels significantly increased the electrical coupling, and that conversely, blockade of astrocytic K_ATP_ channels significantly decreased the electrical coupling. Importantly, when combining activation of mitoK_ATP_ channels in S_cell_ with blockade of mitoK_ATP_ channels in R_cell_, the coupling ratio increased significantly (Fig. 4). This result suggests different activation statuses of mitoKATP may change the direction of electrical coupling between two astrocytes. Since astrocytic gap junctions are important to neuronal networks, this finding indicates that astrocytic networks sense the substrates (K^+^) concentration. These can also change the direction of electrical coupling for brain functional recovery or for information processing under pathologic conditions. Moreover, other metabolic machinery may also contribute to the electrical coupling besides gap junctional coupling and K^+^ buffering.

Although we found that DIZ increased mitochondrial Cx43 and p-ERK expressions and their direct interactions, and demonstrated that inhibition of ERK1/2 could attenuate the effects of DIZ on electrical coupling, suggesting that DIZ regulates astrocytic electrical coupling might via up-regulation of the mitochondrial Cx43-constituted gap junction coupling by an ERK-dependent mechanism. However, we did not provide direct evidence for the involvement of mitochondrial Cx43 in the observed effect of DIZ on electrical coupling, as there is no specific inhibitor of the mitochondrial Cx43 based on the available literature. Availability of animals with ablated astrocytic mitochondrial Cxs and of precisely selective blockers of astrocytic mitochondrial gap junctions might promote a better understanding of the DIZ-induced electrical couplings. In addition, DIZ-induced ERK phosphorylation might enhance electrical coupling through other pathways, because we demonstrated that inhibition of ERK1/2 only could attenuate the effects of DIZ on electrical coupling by 61%. Further studies are needed to clarify these issues.

Taken together, with these findings it is likely that opening of astrocytic mitoK_ATP_ channels by DIZ activates ERK by a ROS-dependent mechanism [58], and increases direct interactions between mitochondrial Cx43 and ERK (Fig. 6), which results in an increased permeability of mitochondrial gap junctions for K^+^. Opening of mitoKATP channels also enhances the mitochondrial K^+^ influx into the soma. Both may increase the K^+^ buffering via sK_ATP_ channels and the membrane gap junction coupling. Activation of astrocytic mitoK_ATP_ channels sustains metabolic machinery and increases the ATP/ADP ratios, which inhibits the inward K^+^ currents of sK_ATP_ channels. In addition, the elevated levels of ATP, proposed to be able to promote astrocytic multiple functions including gap junction coupling, lactate transportation and ion transferring [59], may facilitate the electrical coupling. Further studies are needed to clarify the missing links in the proposed model (Fig. 7).

### Pathophysiological implications and perspectives

Previous pharmacological evidence has suggested that K_ATP_ channel openers are effective anticonvulsants [21]–[24]. However, little is known about the anticonvulsant effect of astrocytic K_ATP_ channel activation, particularly of astrocytic mitoK_ATP_ channels, and the underlying mechanism of this effect. We previously showed that the anticonvulsant action of K_ATP_ channels is partly through the enhancement of the astrocytic mitoK_ATP_ channel-regulated gap junction coupling and the neuronal K_ATP_ channel-modulated excitability [33]. Therefore, our novel finding of astrocytic mitoK_ATP_ channel openers as regulators of electrical coupling may provide a new strategy for anti-epilepsy treatment.

In addition, availability of animals with ablated astrocytic mitoK_ATP_ channels or astrocytic Cxs and of precisely selective openers/blockers of astrocytic mitoK_ATP_ channels or gap junctions might also promote a better understanding of the proposed roles of astrocytic mitoK_ATP_ channel-regulated gap junctional / electrical couplings in glia-related transcellular signalling pathways involved in the physiopathology of the brain.

In summary, the impact of activation of astrocytic mitoK_ATP_ channels on the electrical coupling might involve multiple mechanisms including ion buffering. metabolic machinery, and possibly activation of some compensatory mechanisms.

## Supporting Information

Figure S1 **Variation of interastrocytic distance of directly coupled astrocytes in CA1 stratum radiatum.**
(TIF)

Figure S2 **Electrical coupling ratio progressively declines with increasing interastrocytic distance in P21 astrocytes.**
(TIF)

Figure S3 **The membrane resistances of P21 rats for the group of the interastrocytic distance between 20.1–30 µm and 30.1–40 µm.**
(TIF)

Figure S4 **Absence of detectable electrical coupling in astrocyte–neuron and astrocyte-NG2 glia recording pairs.**
(TIF)

Figure S5 **Resting membrane current in a recorded astrocyte did not change after addition of 100 µM DIZ.**
(TIF)

Table S1 **Criteria to differentiate astrocyte from neuron or NG2 glia in hippocampus.**
(DOC)

Text S1 **Methods for astrocyte cultures and the preparation of membrane and mitochondria fractions.**
(DOC)
