# Supplementary figures and images for: Opening of Astrocytic Mitochondrial ATP-Sensitive Potassium Channels Upregulates Electrical Coupling between Hippocampal Astrocytes in Rat Brain Slices
Source: PLoS One. 2013 Feb 13;8(2):e56605. doi: 10.1371/journal.pone.0056605 (PMC3572089; doi:10.1371/journal.pone.0056605)

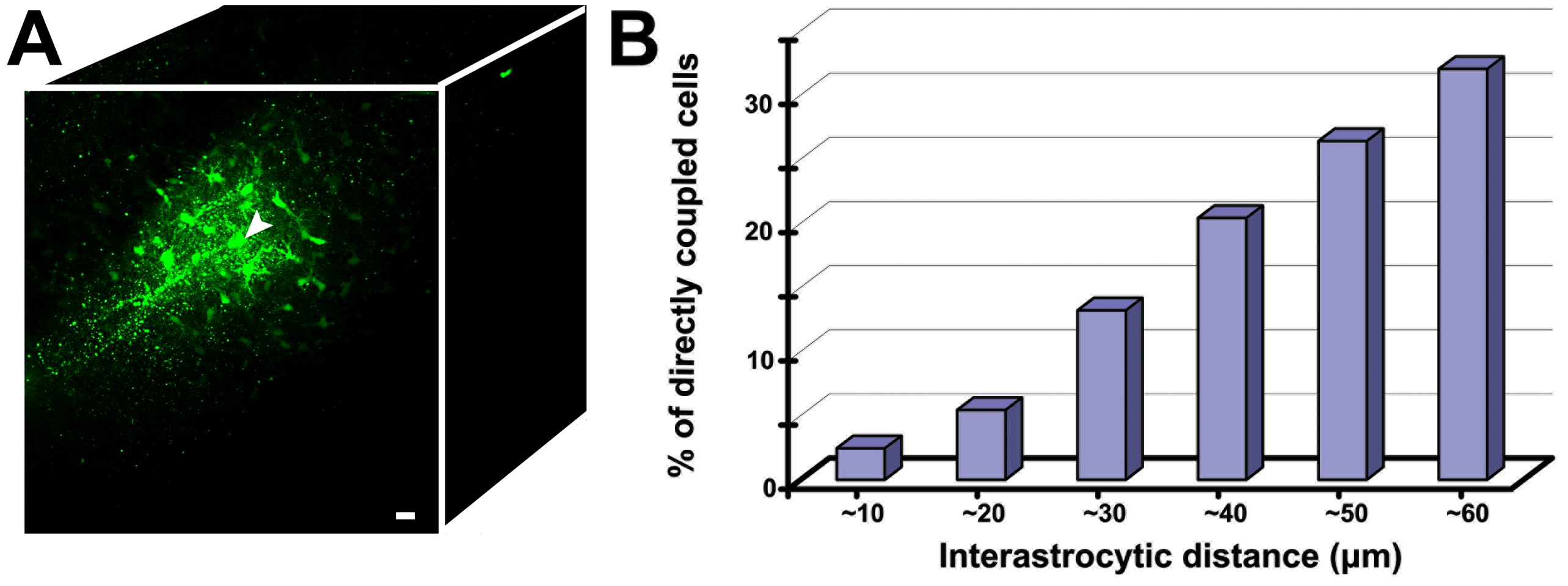

Supplement: Figure S1 — Variation of interastrocytic distance of directly coupled astrocytes in CA1 stratum radiatum. (TIF) [file pone.0056605.s001.tif]

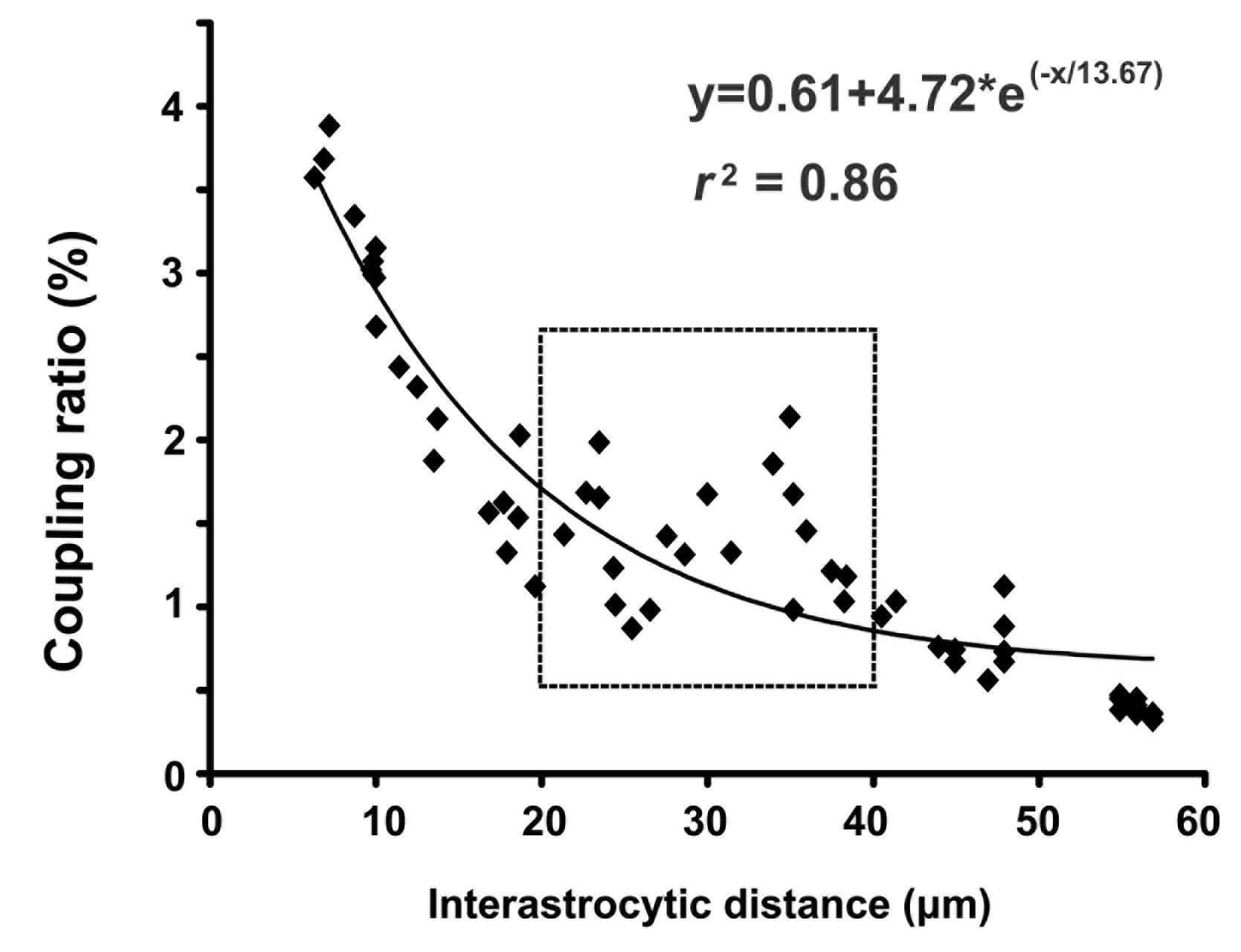

Supplement: Figure S2 — Electrical coupling ratio progressively declines with increasing interastrocytic distance in P21 astrocytes. (TIF) [file pone.0056605.s002.tif]

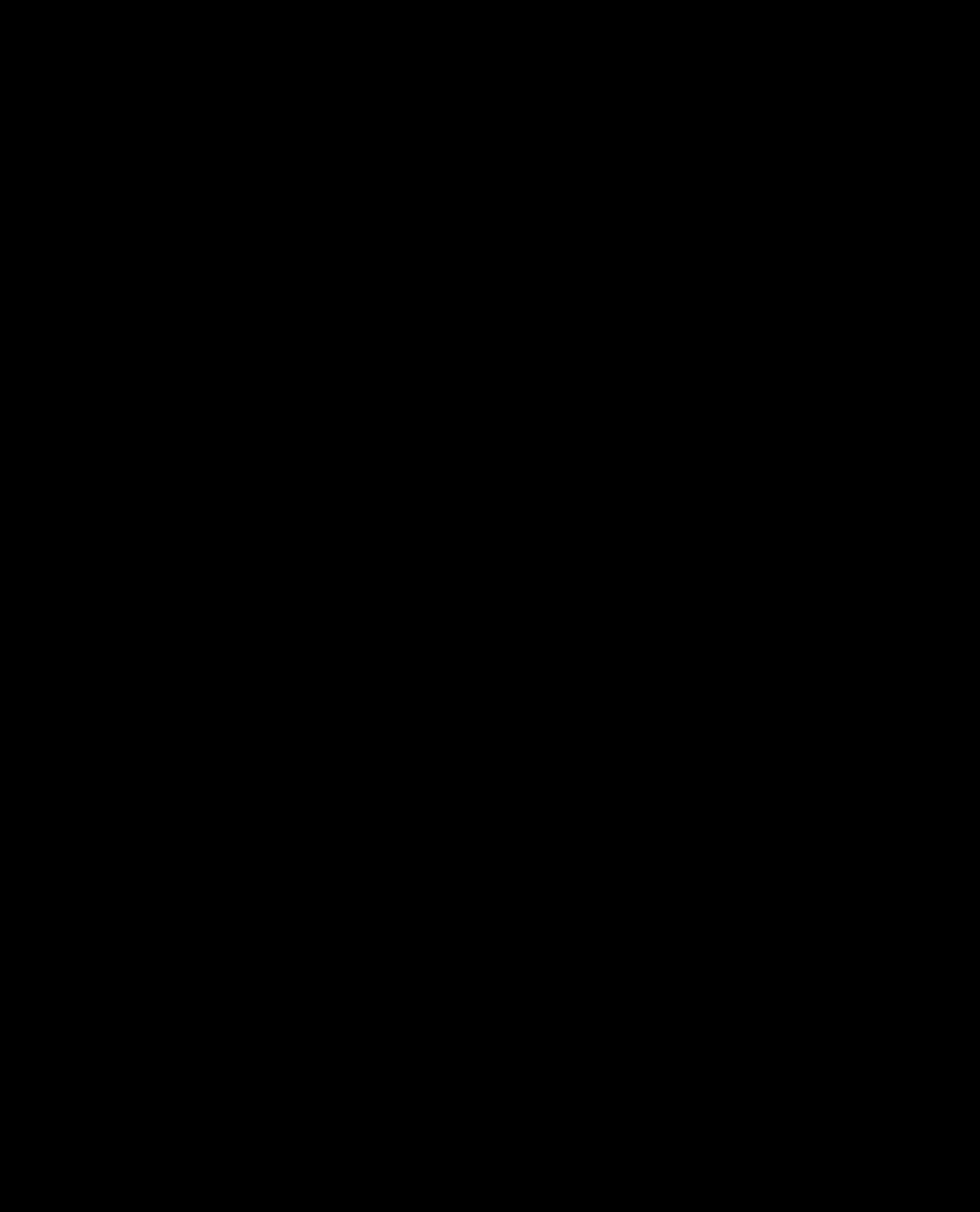

Supplement: Figure S3 — The membrane resistances of P21 rats for the group of the interastrocytic distance between 20.1–30 µm and 30.1–40 µm. (TIF) [file pone.0056605.s003.tif]

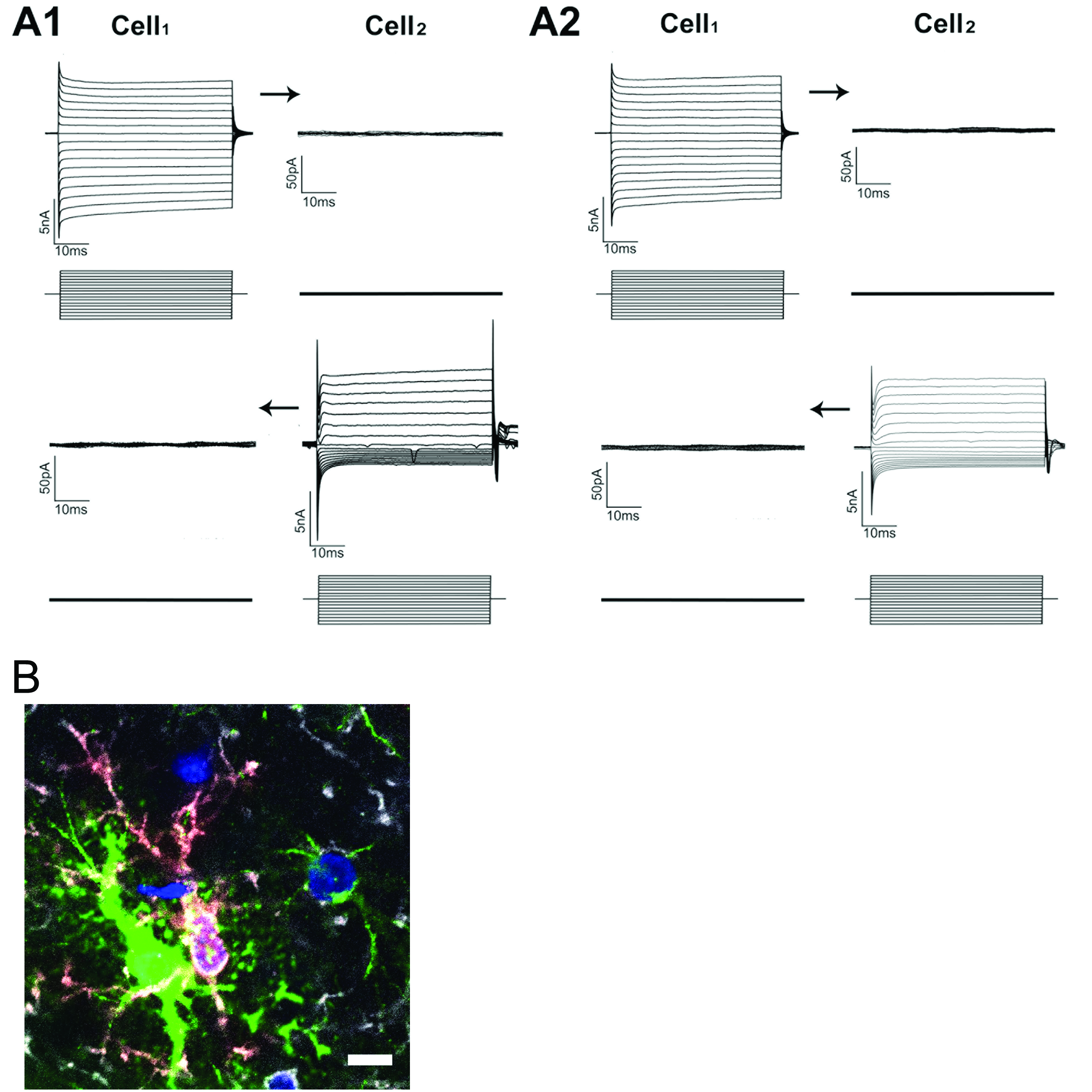

Supplement: Figure S4 — Absence of detectable electrical coupling in astrocyte–neuron and astrocyte-NG2 glia recording pairs. (TIF) [file pone.0056605.s004.tif]

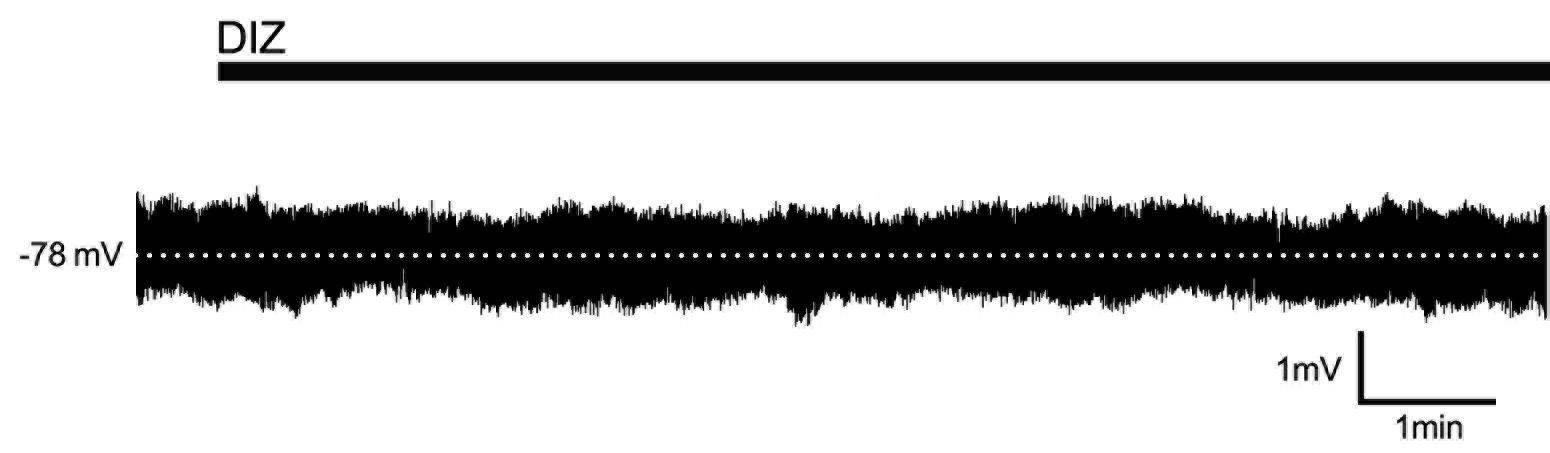

Supplement: Figure S5 — Resting membrane current in a recorded astrocyte did not change after addition of 100 µM DIZ. (TIF) [file pone.0056605.s005.tif]
