# Supplementary material for: Opening of Astrocytic Mitochondrial ATP-Sensitive Potassium Channels Upregulates Electrical Coupling between Hippocampal Astrocytes in Rat Brain Slices
Source: PLoS One. 2013 Feb 13;8(2):e56605. doi: 10.1371/journal.pone.0056605 (PMC3572089; doi:10.1371/journal.pone.0056605)
Supplement: Table S1 — Criteria to differentiate astrocyte from neuron or NG2 glia in hippocampus. (DOC) [file pone.0056605.s006.doc]

Table S1. Criteria to differentiate astrocyte from neuron or NG2 glia in hippocampus

| Cell type | Morphology under IR-DIC | | | | | |
| --- | --- | --- | --- | --- | --- | --- |
|  | Hippocampal location | | Soma diameter(μm) | | Morphologic feature | |
| Pyramidal neuron | Pyramidal neuron layer | | 10-20 | | Round soma with basal dendrite | |
| Interneuron | Stratum radiatum | | >20 | | Irregular or round soma with basal dendrite | |
| Astrocyte | Stratum radiatum | | 5-10 | | Round soma with primary process | |
| NG2 glia | Stratum radiatum | | 5-10 | | Round soma | |
|  | Electrophysiological characteristic | | | | | |
|  | Action potential | Rest membrane potential | | Membrane capacitance (*C_M_*) | | Membrane resistance (*R_M_*) |
| Pyramidal neuron | + | -65-70mV | | 100-200pF | | 50-400 MΩ |
| Interneuron | + | -65-70mV | | 100-200pF | | 50-400MΩ |
| Astrocyte | - | -80mV | | 300-400pF | | 2 MΩ |
| NG2 glia | - | -80mV | | 40-60pF | | 100-200 MΩ |
