# Supplementary material for: Opening of Astrocytic Mitochondrial ATP-Sensitive Potassium Channels Upregulates Electrical Coupling between Hippocampal Astrocytes in Rat Brain Slices
Source: PLoS One. 2013 Feb 13;8(2):e56605. doi: 10.1371/journal.pone.0056605 (PMC3572089; doi:10.1371/journal.pone.0056605)
Supplement: Text S1 — Methods for astrocyte cultures and the preparation of membrane and mitochondria fractions. (DOC) [file pone.0056605.s007.doc]

**Methods**

**Astrocyte cultures**

Astrocyte cultures of rat brain were prepared as described previously . Briefly, forebrains of new born rats (less than 12 h) were removed under sterile conditions, cut into small pieces and dissociated with 0.05% trypsin. After the dissociation procedure, the brain cells were cultures in 25-ml flasks in Eagle’s minimum essential medium, supplemented with 10% horse serum, 2% fetal calf serum, 0.6% glucose, 50 μg/ml gentamycin, and penicillin-streptomycin (5 IU/ml and 5 μg/ml, respectively). Cell cultures were kept at 37ºC in a modified atmosphere of 5% CO_2_ in air. The medium was changed twice a week. Immunocytochemistry verified that the cells stained positively for the astrocytic marker glial fibrillary acid protein (GFAP).

**Preparation of membrane and mitochondria fractions**

Separation of membrane fractions from astrocytic cultures were performed as previously reported. In brief, astrocytes were collected from culture dishes and homogenized in ice-cold Tris buffer containing 20 mM Tris-HCl, 1 mM EGTA, 5 mM NaN_3_, 50 mM NaCl, 1 mM PMSF, 10 mM β-mercaptoptoethanol, 100 mM Na_3_VO_4_ and a protease inhibitor cocktail (Roche Diagnostics). The homogenate was first centrifuged at 600×g for 10 min to separate myofibrils and nuclei, and the supernatant was re-centrifuged at 10,000×g for 20 min. This 10,000×g pellet was used as the membrane fraction. Protein level in the fraction was determined by a Bio-Rad Protein Assay Kit (Bio-Rad).

For mitochondria fractions, astrocytes were homogenized in a Potter-Elvehjem grinder (buffer containing 100 mM KCl, 5 mM MgSO_4_, 50 mM 3-[*N*-morpholino]-propanesulfonic acid [MOPS], 1 mM EGTA, 1 mM MgATP, 0.04% BSA, pH 7.4), and spun at 800 *g* for 10 min. The pellet containing the mitochondria was resuspended in buffer, treated for 1 minute with nargase (8 U/g tissue [bacterial, type XXIV, Sigma-Aldrich]), homogenized with a Potter-Elvehjem grinder, and spun at 800 *g* for 10 minutes. The supernatant was further purified by an additional centrifugation cycle (8,000 *g*, 10 minutes, 4°C) and the final pellet resuspended in K^+^ solution (in mM: 150 KCl, 5 K-HEPES, 1 CaCl_2_, pH 7.2). Mitochondria were stored at -80 °C. Mitochondria protein was prepared by osmotic shock or digitonin as previously described .

**Figure legends**

**Fig. S1.**  Variation of interastrocytic distance of directly coupled astrocytes in CA1 stratum radiatum (SR). (**A**) Two-phton 3D image of an astrocytic syncytium shown by intracellular LY loading (green) of a whole-cell recorded astrocyte (indicated by a white arrow). The 3D image was used for identifying those astrocytes directly coupled to the recorded cell (arrow) and for measuring interastrocytic distance (see Methods section). Scale bar = 10μm. (B) Bar graph shows the percentage distribution of directly coupled astrocytes that fall into different interastrocytic distance ranges in P21-25 rat slice.

**Fig.S2.** Electrical coupling ratio progressively declines with increasing interastrocytic distance in P21 astrocytes. The coupling ratio is the percentage of the I_R_/I_S_ (0-5%), not normalized to the control level. The interastrocytic pair distances are plotted against their corresponding electrical coupling ratios. The data points followed an inverse exponential relationship. The square showing the variant of the coupling ratio was similar within the interastrocytic distance between 20.1-40 μm. The levels between the two groups (interastrocytic distance: 20.1~30 vs. 30.1~40 μm) were not significantly different (*P* > 0.05).

**Fig. S3.** The membrane resistances (Rm) of P21 rats varied from 1.5 to 3.6 MΩ for the group of the interastrocytic distance between 20.1-30 μm, and from 1.1 to 3.8 MΩ for the group of the interastrocytic distance between 30.1-40 μm respectively. There is no significant difference between the two groups (*P* > 0.05). Mean ± SEM.

**Fig. S4.** Absence of detectable electrical coupling in astrocyte–neuron and astrocyte-NG2 glia recording pairs. (A1) Dual patch recordings from the pair of astrocyte (cell1)–neuron (cell2). The recorded neuron (right) showed voltage-gated inward Na^+^ and outward K^＋^ channel currents. No electrical coupling was detected in this astrocyte–neuron pair. (A2) Dual patch recordings from the pair of astrocyte (cell1)–NG2 glia (cell2). The recorded NG2 glia (right) showed voltage-gated outward K^+^ channel currents and small inward Na^+^ currents. No electrical coupling was detected in this astrocyte–NG2 glia pair. (B) Single z-plane confocal image from CA1 stratum radiatum (SR) containing a recorded astrocyte-NG2 glia pair. The recorded astrocyte was filled with LY (green) and NG2 glia was filled with Alexa Fluor® 594 (red) and showed positive NG2 staining (white). DAPI (blue) for nuclear. Scale bar = 10μm.

**Fig. S5.** Resting membrane current in a recorded astrocyte did not change after addition of 100 μM DIZ. We recorded the rest membrane currents using aCSF containing 100 μM-DIZ in an astrocyte and found that DIZ did not affect the rest membrane currents during the 10-min administration. The results indicated that 100μM-DIZ did not act on the membrane K_ATP_ channels whose activation would result in the hyperpolarization of the cell. n = 5.

1. Weinstein, D.E., *Isolation and purification of primary rodent astrocytes.* Curr Protoc Neurosci, 2001. **Chapter 3**: p. Unit 3 5.

2. Zhang, S., et al., *ATP-sensitive potassium channel opener iptakalim protects against MPP-induced astrocytic apoptosis via mitochondria and mitogen-activated protein kinase signal pathways.* J Neurochem, 2007. **103**(2): p. 569-79.

3. Naitoh, K., et al., *MitoKATP channel activation suppresses gap junction permeability in the ischemic myocardium by an ERK-dependent mechanism.* Cardiovasc Res, 2006. **70**(2): p. 374-83.

4. Jiang, K., et al., *Regulation of gap junctional communication by astrocytic mitochondrial K(ATP) channels following neurotoxin administration in in vitro and in vivo models.* Neurosignals, 2011. **19**(2): p. 63-74.

5. Inoue, I., et al., *ATP-sensitive K+ channel in the mitochondrial inner membrane.* Nature, 1991. **352**(6332): p. 244-7.

Table

Table S1. Criteria to differentiate astrocyte from neuron or NG2 glia in hippocampus

| Cell type | Morphology under IR-DIC | | | | | |
| --- | --- | --- | --- | --- | --- | --- |
|  | Hippocampal location | | Soma diameter(μm) | | Morphologic feature | |
| Pyramidal neuron | Pyramidal neuron layer | | 10-20 | | Round soma with basal dendrite | |
| Interneuron | Stratum radiatum | | >20 | | Irregular or round soma with basal dendrite | |
| Astrocyte | Stratum radiatum | | 5-10 | | Round soma with primary process | |
| NG2 glia | Stratum radiatum | | 5-10 | | Round soma | |
|  | Electrophysiological characteristic | | | | | |
|  | Action potential | Rest membrane potential | | Membrane capacitance (*C_M_*) | | Membrane resistance (*R_M_*) |
| Pyramidal neuron | + | -65-70mV | | 100-200pF | | 50-400 MΩ |
| Interneuron | + | -65-70mV | | 100-200pF | | 50-400MΩ |
| Astrocyte | - | -80mV | | 300-400pF | | 2 MΩ |
| NG2 glia | - | -80mV | | 40-60pF | | 100-200 MΩ |
